# Different DNA input amounts and library preparation kits yield comparable resistome characterization using target-enriched metagenomic sequencing

**DOI:** 10.3389/fmicb.2026.1907850

**Published:** 2026-09-10

**Authors:** Enrique Doster, Lee J. Pinnell, Jennifer K. Parker, Cory A. Wolfe, Paul S. Morley

**Affiliations:** 1 VERO Program, Texas A&M University, Canyon, TX, United States; 2 Department of Clinical Sciences, Colorado State University, Fort Collins, CO, United States

**Keywords:** antimicrobial resistance, antimicrobial resistance genes, ARGS, resistome, microbial ecology, microbiome, probe capture, sequencing methods

## Abstract

**Introduction:**

Target-enriched metagenomic sequencing improves detection of antimicrobial resistance genes (ARGs) relative to standard shotgun metagenomics, but whether DNA input amount influences resistome characterization has not been systematically evaluated.

**Methods:**

We evaluated target-enriched sequencing using fecal microbial communities from multiple host species and compared results across library preparation kits, DNA input amounts, and sequencing approaches. Composite fecal samples from livestock and humans and individual canine samples were processed using three library preparation kits and DNA input amounts ranging from 50 to 800 ng.

**Results:**

Target-enriched libraries from composite samples were compared with shotgun metagenomic sequences generated from the same samples. Target-enriched sequencing achieved a 7.9-fold increase in on-target read proportions and detected 12-fold more unique antimicrobial resistance gene groups than shotgun metagenomics. Resistome composition was driven primarily by sample source, which explained 66%–79% of within-species variance, while library preparation kit accounted for 13%–26% and DNA input amount had no consistent effect. Kit-associated differences were systematic and affected primarily low-abundance resistance classes but were small relative to biological variation among samples.

**Discussion:**

These findings demonstrate that target-enriched metagenomic sequencing substantially improves resistome detection and provides comparable resistome characterization DNA input amounts ranging from 50 to 800 ng. Differences associated with library preparation were small relative to biological variation, supporting the flexibility of using differing DNA inputs for resistome characterization.

## Introduction

Shotgun metagenomic sequencing is a powerful approach for characterizing complex microbial communities across diverse environments, from gastrointestinal ecosystems to soil. Characterizing genomic content from entire microbial populations enables comprehensive analysis of microbial taxa and determinants of their functional potential, such as antimicrobial resistance (AMR) genes. However, the accuracy and reliability of shotgun metagenomic analysis depend heavily on the specific laboratory and analytical workflows, including DNA extraction, library preparation, and sequencing (Kircher and Kelso, 2010; Henderson et al., 2013; Head et al., 2014; Zaheer et al., 2018; Pinnell et al., 2024). Additionally, shotgun sequencing can be inefficient when the objective is to characterize low-abundance features, such as antimicrobial resistance genes (ARGs), or when large amounts of off-target DNA, such as host DNA, are present. Because AMR genes often comprise ≤0.1% of shotgun sequencing reads in complex bacterial communities such as feces, achieving sufficient sequencing depth for detailed resistome characterization can be costly and technically challenging, particularly for samples with high background DNA or complex matrices such as food products (Noyes et al., 2017; Guitor et al., 2019; Doster et al., 2020).

One strategy for improving the efficiency of shotgun sequencing is to enrich specific genomic targets before sequencing. One method for target-enriched (TE) sequencing uses probes that are complementary to the genomic targets to capture and enrich specific DNA sequences during library preparation, increasing the proportion of informative reads in the final sequencing output. Previous studies have demonstrated the utility of TE sequencing for investigating antimicrobial resistance (AMR) and a variety of specific targets in complex microbial communities using both short- and long-read sequencing (Noyes et al., 2017; Guitor et al., 2019; Allicock et al., 2018; Doster et al., 2022; Pinnell et al., 2023; Slizovskiy et al., 2024). What remains unresolved is whether the resistome profiles generated by these approaches are stable across the range of laboratory choices available to investigators, including DNA input amount and library preparation kit, which determines whether results from different studies can be meaningfully compared. Multiple commercial platforms now support customizable TE sequencing approaches, and probe sets such as MEGaRICH have been developed to enable enrichment of comprehensive collections of ARGs derived from all published AMR genes as compiled in the MEGARes databases^1^ (Noyes et al., 2017; Doster et al., 2020; Bonin et al., 2022). These probe-enrichment approaches offer flexibility in laboratory workflows, including up to 100-fold differences in DNA input amounts, hybridization conditions, amplification parameters, and protocol duration. Higher DNA inputs may increase representativeness of sampled DNA, whereas lower-input protocols can reduce costs and enable analysis of samples with limited material. Determining whether these differing strategies yield comparable resistome profiles is therefore critical for optimizing study design and ensuring comparability across investigations.

**Figure 1:**
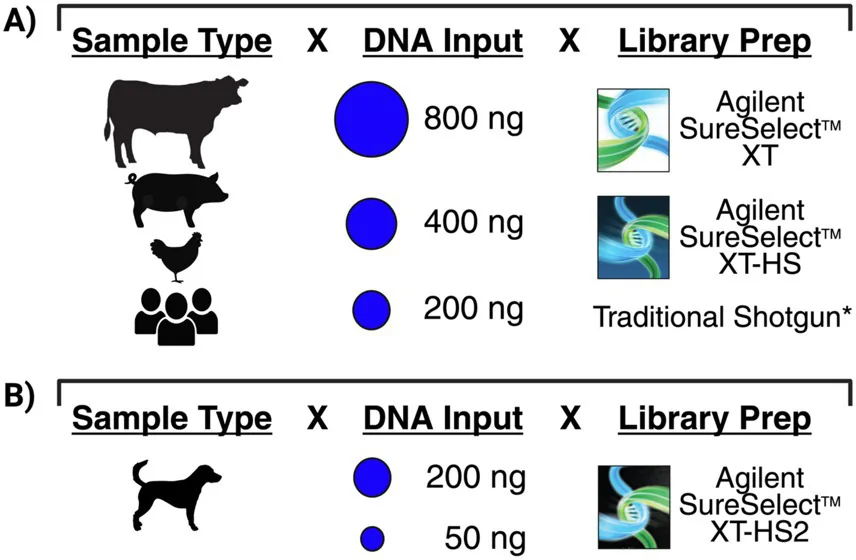
Overview of study design. The comparability of short-read TE shotgun sequencing of AMR genes was evaluated using commercially available library enrichment and preparation kits and shotgun metagenomic sequencing. **(A)** Three DNA input amounts (200 ng, 400 ng, and 800 ng) were used for fecal samples from four host species (humans, cattle, pigs, and chickens), each process with two target-enrichment library preparation kits (SureSelect XT and XT-HS). *DNA input was not varied for traditional shotgun sequencing. **(B)** Fecal samples from dogs were analyzed using two DNA input amounts (50 ng and 200 ng) with the SureSelect XT-HS2 kit. All target-enrichment libraries were prepared using the same custom probe set and sequenced using Illumina short-read (2 × 150 bp) platforms. This figure was created in biorender.com.

In this study, we evaluated the comparability of short-read target-enriched metagenomic sequencing for characterization of antimicrobial resistance genes with four different DNA input amounts and two library preparation kits using fecal microbial communities from five host species (humans, chickens, cattle, pigs, and dogs—Figure 1). Additionally, we directly compared TE sequencing to standard shotgun metagenomics using matched samples to quantify the advantage of TE for resistome characterization. The goal of this research was to determine whether TE sequencing provides consistent and reliable resistome profiles across different laboratory protocols and sample types. Fecal communities from multiple hosts were used as complex, biologically realistic matrices against which the consistency of different library preparation conditions could be evaluated.

## Materials and methods

### Study overview

This study evaluated target-enriched (TE) sequencing for characterizing antimicrobial resistance genes (ARGs) in fecal samples. We investigated how resistome profiles varied by DNA input amount and library preparation protocol (Parts A and B) and compared TE sequencing to shotgun metagenomic sequencing (Part A). The study was divided into two parts: Part A analyzed composite fecal samples from beef cattle, pigs, chickens, and humans (*n* = 4 per species; Panel A—Figure 1), while Part B analyzed individual fecal samples from dogs in two different countries (*n* = 8 per country, Panel B—Figure 1). All TE libraries (Part A *n* = 24; Part B *n* = 16) were sequenced on Illumina platforms (2 × 150 bp).

### Study population and sample collection

Composite fecal samples analyzed in Part A were previously described (Noyes et al., 2017) and are briefly summarized here. Samples were collected from animals housed in four different housing groups across different operations (four pens of feedlot cattle in Arizona, four pens of finishing pigs in Colorado, and four houses of broiler chickens in Florida). Twenty fresh fecal samples were collected from the floors of each pen/house and composited by housing group (*n* = 20 samples per composite and *n* = 4 composite samples per species). Additionally, four large volume (~100 mL) samples were collected from a municipal sewage treatment plant in Arizona to represent composited samples of human waste. All samples were placed on ice immediately after collection and shipped the same day to the laboratory where they were frozen until processed. For the current study, archived aliquots from these original samples were processed for target-enriched (TE) sequencing. We also downloaded the shotgun metagenomic sequences from the corresponding samples (Noyes et al., 2017) and re-analyzed them alongside the TE data to enable direct comparison between the two sequencing approaches (NCBI Bio Project PRJNA339554). Fecal samples used in Part B were collected from healthy dogs living in different households in the UK and Portugal (*n* = 4 per country, total *n* = 8) (manuscript *under-review*).

### DNA isolation, target enrichment, and library preparation

*Part A*: DNA was extracted from composite fecal samples collected using a commercial kit (DNeasy® PowerMax Soil Kit, Qiagen, Hilden, Germany), following the manufacturer’s instructions with the following modifications: Approximately 10 g of samples were used as input for the DNA extraction process, and samples were lysed before extraction using bead beating (Mini-BeadBeater-24, BioSpec Products, Bartlesville, OK) with five 1-min pulses (3,450 rpm) and 5-min rests between each pulse. Multiple extractions were conducted for all samples and pooled to obtain ≥2.8 μg of purified DNA per sample for downstream analysis. Recovered DNA was quantified (ng/μL) using fluorometry (Qubit 4, Thermo Fisher Scientific). Multiple aliquots of pooled DNA for each sample were acoustically sheared (M220 Focused-ultrasonicator, Covaris, LLC, Woburn, MA) with 8.7–12 ug of DNA per sample with 2–7 rounds of shearing, depending on sample concentration, for 100 s per round with 75 W treatment power, 20% duty factor, and 200 cycles/burst. The sheared product was size-selected and cleaned using solid-phase reversible immobilization (SPRI) beads (Mag-Bind TotalPure NGS, Omega Bio-tek, Norcross, GA, USA) at a 0.8 × bead-to-sample ratio to retain fragments >250–300 bp and then pooled by sample for automated electrophoresis analysis (4,150 TapeStation System, Agilent Technologies, Santa Clara, CA) to confirm a target insert size.

Target enrichment and library preparation were conducted for all samples using technical replicates of the sheared, purified DNA for each of the six conditions: three different DNA input amounts (800 ng versus (vs.) 400 ng vs. 200 ng) and two different commercial kits for processing (SureSelect™ XT vs. SureSelect™ XT-HS, Agilent Technologies, Santa Clara, CA, USA) according to the manufacturer’s recommendations, with the following conditions: For each of the three DNA input amounts, eight PCR cycles were used during end repair and A-tailing, and 750 ng of product was passed into hybridization. The previously described MEGARich probe set (Noyes et al., 2017; Lakin et al., 2017) that was based on the MEGARes v1.0 comprehensive database of ARGs, which included 3,824 published ARG sequences, was included in a 90-min hybridization step, followed by 15 cycles of post-capture PCR. TE Libraries were characterized using automated electrophoresis analysis (4,150 TapeStation System, Agilent Technologies, Santa Clara, CA) and pooled in equimolar amounts for sequencing on the Illumina HiSeq 1,500 sequencer using 2 × 150 bp paired-end chemistry at a commercial laboratory (Novogene, Durham, NC). The same pool was sequenced on six lanes to obtain a targeted average sequencing depth of 20 M paired-end reads per sample.

*Part B*: DNA was extracted using a commercial kit (DNeasy® PowerSoil Kit, Qiagen, Hilden, Germany) following the manufacturer’s instructions with the following modifications: Approximately 200 mg samples were used as input for the DNA extraction process and were lysed before extraction using bead beating as described above. Multiple extractions were conducted when necessary to provide a total of 2 μg of purified DNA. The recovered DNA was processed as described above before library preparation.

Target enrichment and library preparation were conducted for Part B fecal samples using technical replicates of the sheared, purified DNA, comparing two different DNA input amounts (200 ng vs. 50 ng) using a similar commercial kit (SureSelect™ XT-HS2, Agilent Technologies, Santa Clara, CA, USA) according to the manufacturer’s recommendations, with the following conditions: For each of the two DNA input amounts, seven PCR cycles were used during end repair and A-tailing, and 1.0 μg of product was passed into hybridization. An updated version of the previously described MEGARich probe set, based on the MEGARes v2.0 comprehensive database of ARGs, which included 7,868 published ARG sequences, was included in a 90-min hybridization step, followed by 16 cycles of post-capture PCR. TE Libraries were characterized using automated electrophoresis analysis (4,150 TapeStation System, Agilent Technologies Santa Clara, CA). Libraries were pooled with samples for another project in equimolar amounts for sequencing on a single NovaSeq 6,000 S4 lane (Illumina, San Diego, CA, USA) using 2 × 150 bp paired-end chemistry at the University of Colorado Genomics Shared Resource Facility (Aurora, CO) to obtain a targeted average sequencing depth of 32 M paired-end reads per sample.

### Bioinformatic processing

Shotgun metagenomic sequences from Part A were downloaded from NCBI SRA using the SRA Toolkit (Leinonen et al., 2011). The shotgun sequences and TE libraries from Part A (Supplementary files 1, 2), as well as the TE libraries from Part B (Supplementary file 3), were all analyzed in the same manner as described below. Raw metagenomic sequencing reads were analyzed to characterize the resistome using the AMR++ bioinformatic pipeline and the MEGARes v3 database (Bonin et al., 2022). In brief, AMR++ uses fastQC (Andrews, 2010) and multiQC (Ewels et al., 2016) to evaluate read quality scores and then employs Trimmomatic (Bolger et al., 2014) for QC trimming. Trimmed reads were then aligned using BWA (Li, 2013) to the MEGARes v3 database. To account for potential PCR bias, alignments were deduplicated using samtools’ *markdup* function (Li et al., 2009). AMR++ then applies a “gene fraction” threshold to retain counts only for gene accessions where at least one read aligns across 80% of the reference sequence. Finally, alignments to gene accessions that require the presence of particular single-nucleotide polymorphism (SNP) to confer resistance were further processed to confirm the presence of these SNPs using the AmrPlusPlus_SNP software^2^. SNP-confirmed counts were used for all downstream analyses.

### Statistical analysis

We used the R programming language for data handling and statistical comparison. For Part A samples, we examined primary comparisons of interest, namely, between library preparation kit (XT vs. XT-HS), DNA input amount (200 ng, 400 ng, 800 ng), sequencing method (TE vs. shotgun), and sample host species. For Part B, the main comparison of interest was between DNA input amounts. The resistome count matrix was loaded using the “phyloseq” R package (McMurdie and Holmes, 2013).

To characterize alpha diversity, the estimate_richness() function was used to calculate richness as the number of observed AMR gene accessions and evenness was estimated using Shannon’s index. Sequencing performance, on-target sequencing counts, and diversity values were statistically compared between sample groups using the Kruskal–Wallis function, “kruskal.test().” Significant comparisons were followed by a pairwise Wilcoxon tests with the “pairwise.wilcox.test()” function and adjustment for multiple comparisons using the Benjamini–Hochberg procedure. Cumulative sum scaling (CSS) as implemented by the “metagenomeSeq” R package (Paulson et al., 2013) was used to normalize counts for resistome composition visualization and beta diversity calculations. CSS-normalized counts were Hellinger-transformed, and beta diversity was measured using Euclidean distances as this approach reduces the influence of highly abundant features and makes Euclidean-based ordination methods appropriate for compositional datasets (Legendre and Gallagher, 2001).

Euclidean distances were computed using the vegdist() and decostand() functions from the vegan package (Oksanen et al., 2014). All beta diversity analyses were performed separately for Dataset A (bovine, avian, porcine, and human wastewater samples) and Dataset B (canine samples). For Dataset A, an initial marginal permutational multivariate analysis of variance (PERMANOVA) model including both host group and original sample source as predictors revealed perfect collinearity between these variables; host group membership was fully defined by the combination of original sample identities, resulting in zero degrees of freedom for the interaction term. Consequently, original sample source was retained as the primary grouping variable in all subsequent models, as it captured the maximum explainable variance. Marginal PERMANOVA models (adonis2(), by = “margin,” 9,999 permutations) were then fit to the full Dataset A distance matrix with original sample source, library preparation kit, and DNA input amount as predictors. Because original sample source explained the dominant proportion of variance and was perfectly collinear with host group, per-species subgroup analyses were conducted by extracting the relevant submatrix from the full Dataset A distance matrix for each host species (bovine, avian, porcine, and human wastewater) and fitting the same marginal PERMANOVA model within each subgroup. For Dataset B, marginal PERMANOVA models included original sample source and DNA input amount as predictors. Homogeneity of multivariate dispersion was assessed for all significant PERMANOVA results using betadisper() and permutest() (9,999 permutations) from the vegan package to determine whether significant PERMANOVA results reflected true centroid differences or differences in group dispersion. Two rarefaction analyses were performed. To evaluate whether beta diversity results were influenced by sequencing depth among TE libraries, all analyses were repeated after rarefying counts to the minimum observed depth among TE samples (127,467 reads) using rarefy_even_depth(). Separately, to evaluate whether the detection advantage of TE over shotgun sequencing reflected the greater sequencing depth of TE libraries, alpha diversity was recalculated after rarefying both TE and shotgun resistome count matrices to a common depth of 6,642 AMR gene alignments, the lowest depth observed among shotgun libraries (Supplementary Figure S1). Rarefied analyses used identical statistical workflows to the CSS-normalized analyses; CSS results are reported as primary findings, with rarefied results presented to confirm robustness.

Redundancy analysis was undertaken with the *rda()* function from “vegan,” with the *step()* and *anova()* functions used with 1,000 permutations to identify significant variables for inclusion in the model. The “UpSetR” package was used to visualize set intersections between sample groups. For differential abundance testing of resistome features based on the library preparation kit, the Analysis of Compositions of Microbiomes with Bias Correction (ANCOM-BC) model was used with the ancombc2() R function (Lin and Peddada, 2020), controlling for DNA input, host species, and original sample source.

## Results

### Target-enriched sequencing yielded higher read counts than shotgun metagenomic sequencing

Read quality was excellent with an average Phred quality score >35 across all samples. A single bovine fecal sample processed with the SureSelect XT kit and 400 ng of input DNA failed sequencing and was therefore excluded from downstream analysis. TE libraries yielded a mean of 30.5 million reads (range: 20.1–52.5 M, standard deviation: 4.8 M) in Part A and 31.8 million reads (range: 15.3–84.6 M standard deviation: 16 M) in Part B. Shotgun metagenomic sequencing from Part A yielded significantly fewer reads than TE libraries (mean 18 M read [range: 7.1–27.6 M, standard deviation: 6.5 M] vs. 30.5 M; Wilcoxon rank-sum test, *p* < 0.001). Within TE libraries from Part A, raw read counts did not differ significantly across host, library type, kit, or DNA input amount (*p* > 0.10, Kruskal–Wallis tests). Similarly, shotgun read counts from Part A did not differ by sample group (*p* = 0.54) but had significantly fewer reads compared to TE libraries in Part A (Wilcoxon rank-sum test, *p* < 0.001). TE libraries in Part B showed no significant difference in read counts between DNA input amounts (*p* = 0.75).

### Target enrichment increased on-target reads and performed consistently across kits and DNA inputs

**Figure 2:**
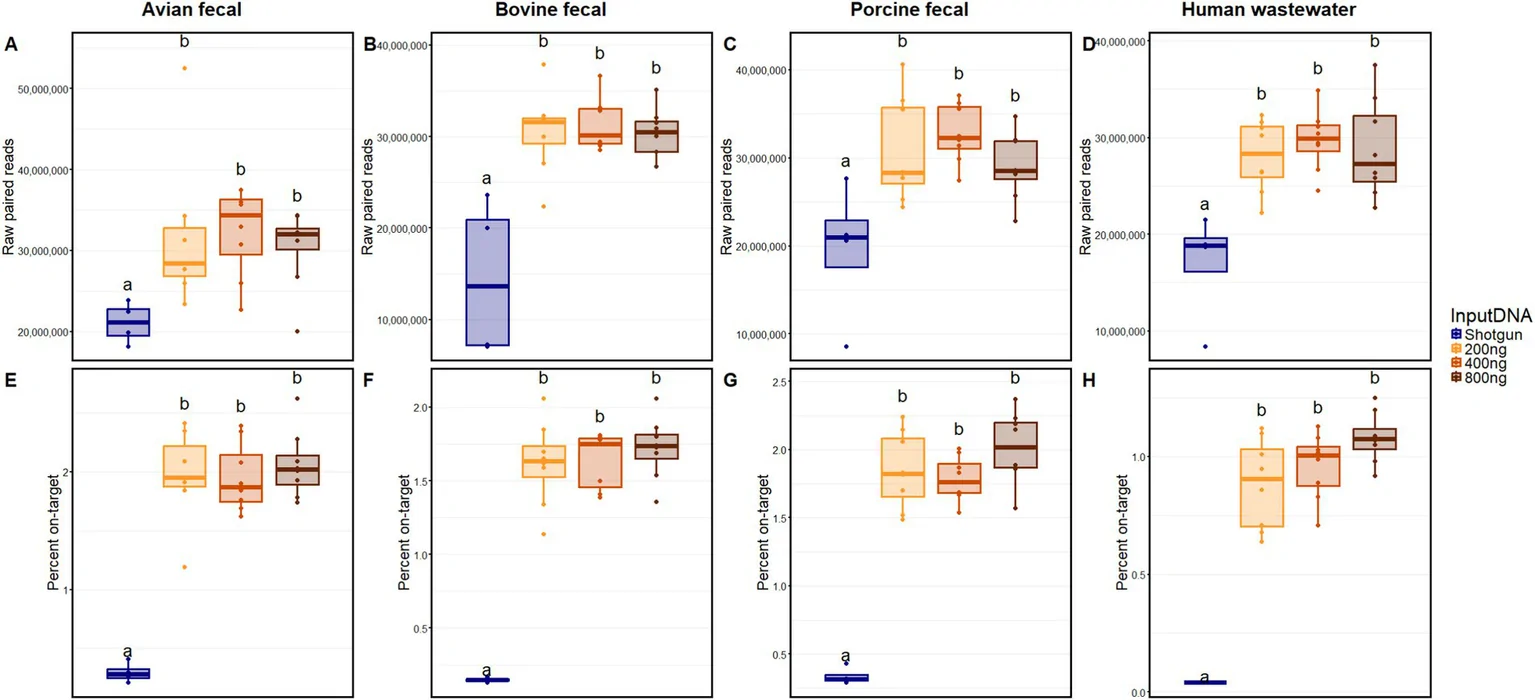
Sequencing performance and on-target enrichment in Part A samples by host source and sequencing method. **(A–D)** Raw paired-end read counts by sample group (avian fecal, bovine fecal, porcine fecal, and human wastewater) and DNA input amount. Shotgun metagenomic libraries (blue) yielded significantly fewer reads compared to target-enriched (TE) libraries across all sample types. **(E–H)** On-target proportion (reads aligning to MEGARes database) by sample group and DNA input amount. TE libraries (200 ng, 400 ng, and 800 ng) achieved substantially higher on-target proportions compared to shotgun sequencing in all host species. Letters above boxplots indicate the results of pairwise Wilcoxon rank-sum tests with Benjamini–Hochberg correction for multiple comparisons, performed within each (sample group/host species). Groups that share a letter are not significantly different (adjusted *p* ≥ 0.05); groups that share no letter differ significantly (adjusted *p* < 0.05). On-target proportions did not differ significantly by DNA input amount within TE libraries for any sample type. Note the different y-axis scales across panels reflecting variation in on-target rates between host species.

**Figure 3:**
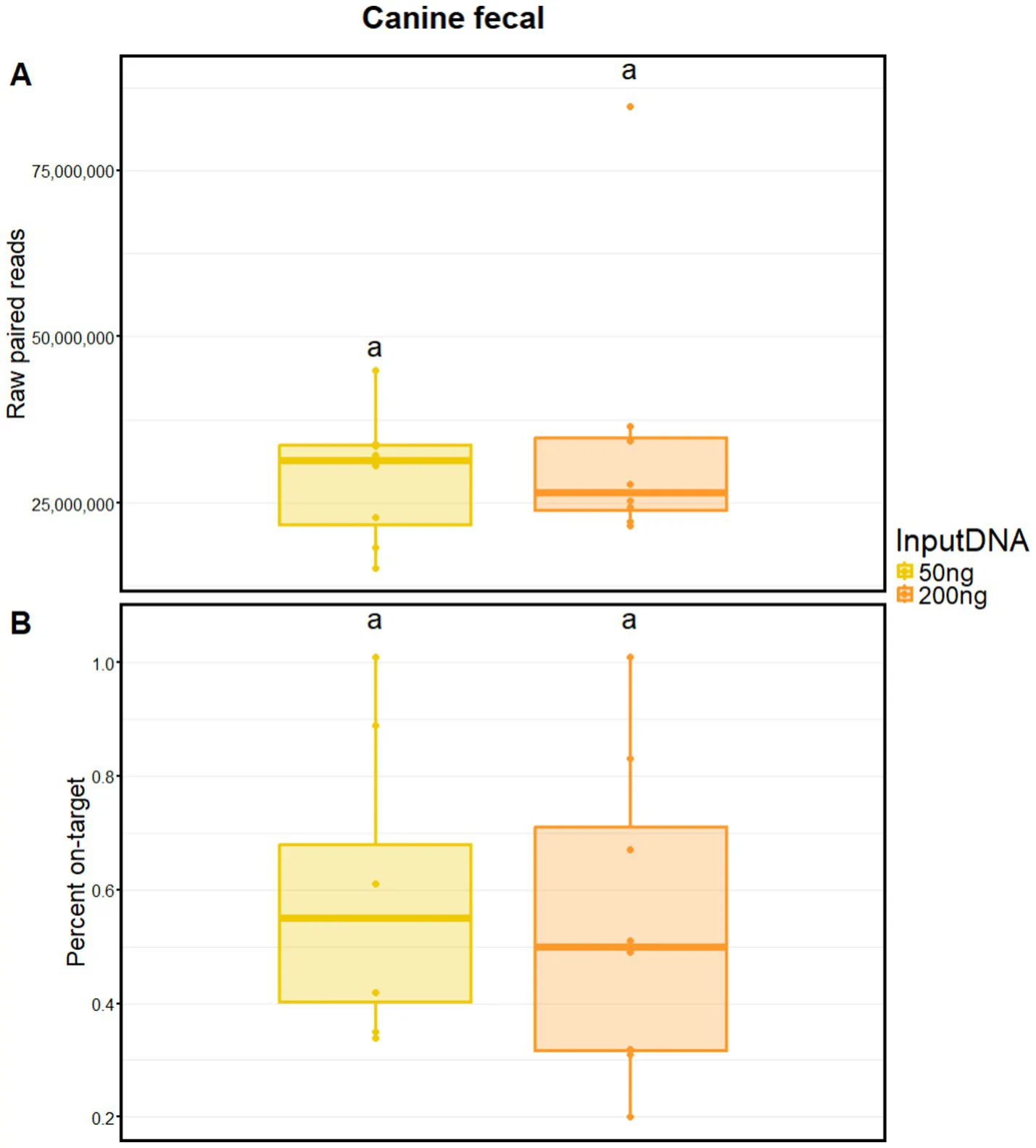
Sequencing performance for Part B canine fecal samples, by DNA input amount (50 ng vs. 200 ng). **(A)** Boxplots showing the numbers of raw paired-end sequencing reads. **(B)** Boxplots showing the proportion of sequencing reads aligning to the MEGARes database by DNA. Letters above boxplots indicate the results of pairwise Wilcoxon rank-sum tests with Benjamini–Hochberg correction for multiple comparisons. Groups that share a letter are not significantly different (adjusted *p* ≥ 0.05); groups that share no letter differ significantly (adjusted *p* < 0.05). No significant difference was observed between input amounts (*p* = 0.46, Wilcoxon rank-sum test).

Following alignment deduplication and SNP confirmation, we identified a total of 98,560,923 hits or alignments to the resistome using the AMR++ pipeline with an average of 782,230 hits per sample (range: 6642–1,379,975, standard deviation: 422,439) (Supplementary files 1–3). Hits to gene accessions requiring SNP confirmation only made up 4.5% of all reads in the resistome. Target-enriched (TE) sequencing achieved significantly higher on-target proportions compared to shotgun metagenomics. In Part A, TE libraries had a mean on-target proportion of 1.62% (range: 0.64%–2.62%, standard deviation: 0.64%), representing a 7.9-fold enrichment compared to shotgun sequencing (mean: 0.21%, range: 0.04%–0.43%, standard deviation: 0.13%; Wilcoxon rank-sum test, *p* < 0.001; Figure 2). On-target proportions varied significantly by sample group for both TE (Kruskal–Wallis, *p* < 0.001) and shotgun (*p* = 0.005) libraries, with human wastewater samples showing the highest on-target rates in TE libraries and the lowest in shotgun libraries. Importantly, on-target proportions in Part A did not differ significantly by library preparation kit (*p* = 0.93), library type (*p* = 0.93), or DNA input amount (*p* = 0.32), indicating robust performance across protocol variations. Similarly, in Part B, on-target proportions (mean: 0.57%, range: 0.20%–1.01%, standard deviation: 0.25%) did not differ significantly between DNA input amounts of 200 ng vs. 50 ng (*p* = 0.46), suggesting that TE library preparation performs consistently even with reduced starting material (Figure 3).

#### Target enrichment detected substantially more AMR gene groups and higher richness than shotgun sequencing

**Figure 4:**
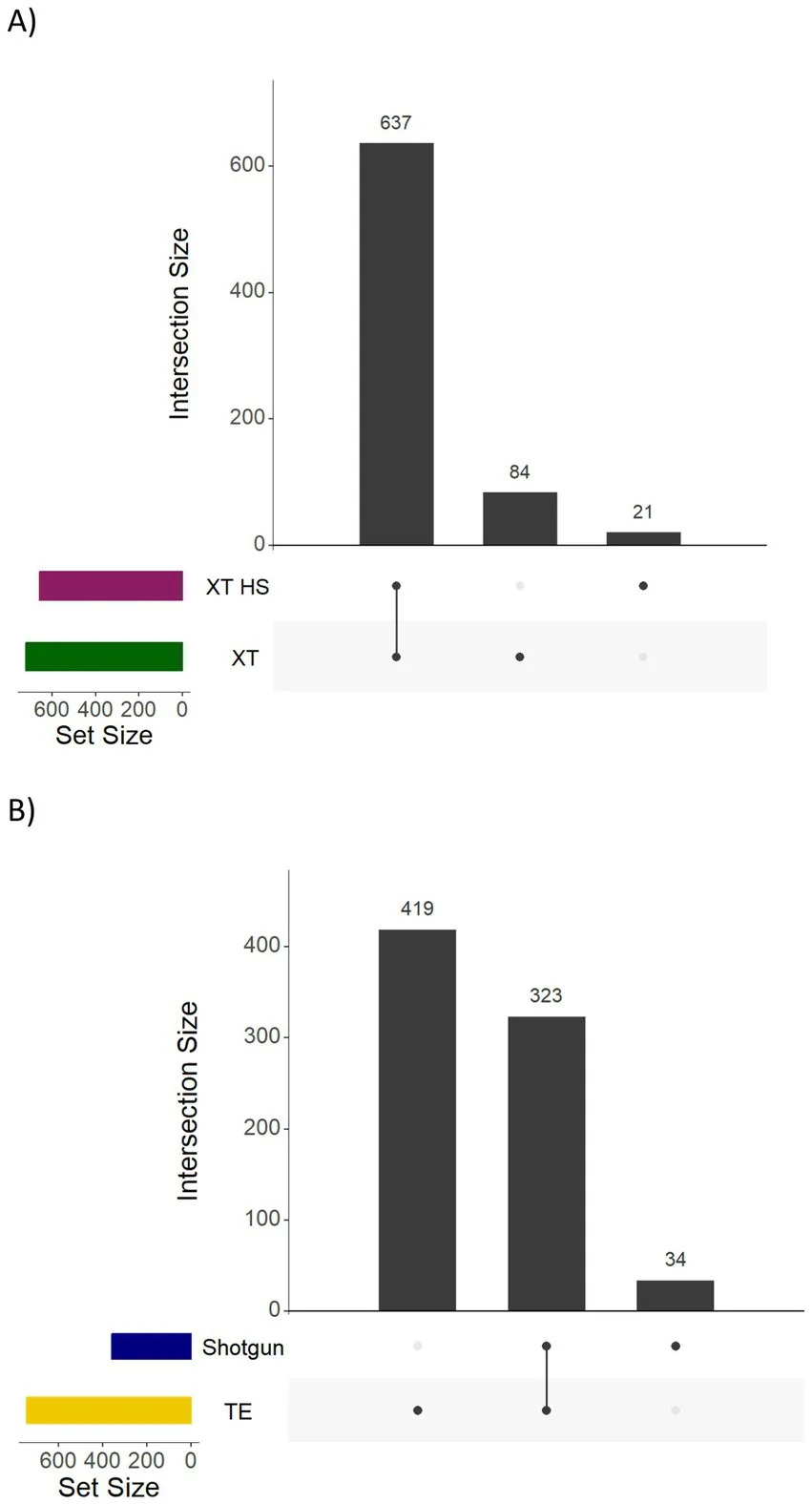
Intersection of AMR gene groups between sequencing methods. **(A)** UpSet plot showing the intersection of AMR gene groups in Part A TE samples by library preparation kit. AMR groups were filtered to include only those present in ≥5% of samples within each host species. The majority (75%) of gene groups were detected by both kits. **(B)** UpSet plot comparing all AMR gene groups detected by shotgun vs. target-enriched (TE) sequencing in Part A samples. TE sequencing detected substantially more unique gene groups (*n* = 419) compared to shotgun sequencing (*n* = 34), with 323 groups shared between methods.

**Figure 5:**
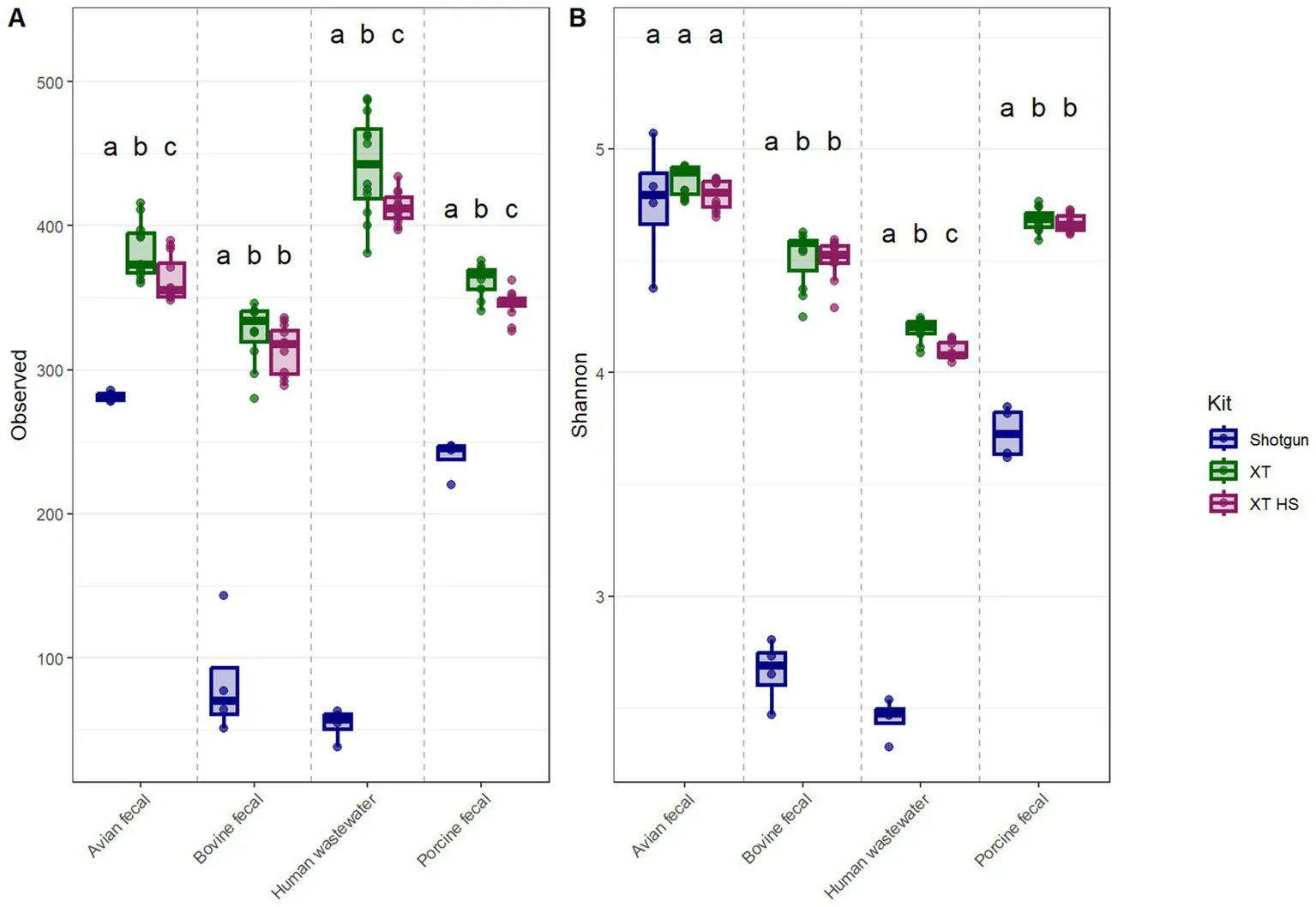
**(A)** Boxplot of unique observed ARGs (richness) on the *y*-axis, grouped by sample host source and library type. **(B)** Boxplot of Shannon’s diversity on the *y*-axis, grouped by sample host source and library kit type. Letters above the boxplots indicate the results of pairwise Wilcoxon rank-sum tests with Benjamini–Hochberg correction for multiple comparisons, performed within each (sample group/host species). Groups that share a letter are not significantly different (adjusted *p* ≥ 0*.05*); *group*s that share no letter differ significantly (adjusted *p* < 0.05).

Across all samples, a total of 2,964 AMR gene accessions were identified, representing resistance to 52 unique drug classes, through 174 resistance mechanisms, and 762 gene groups. With the main comparison in Part A samples being between the SureSelect XT and XT HS target-enrichment kits, the intersection between identified gene groups showed that 75% of all identified gene groups (637/847 groups) were present in both library kits. After applying a filter to only include gene groups present in at least 5% of the samples in each host species, the percentage of shared gene groups increased to 88% (636/719). The SureSelect XT kit picked up an additional 56 gene groups, while the XT HS kit had 27 unique gene groups (Figure 4—Panel A). In Part A samples, 41% of AMR gene groups were shared between TE and shotgun sequencing (323/796), with a larger number detected only by TE (*n* = 419) and relatively few detected only by shotgun (*n* = 34) (Figure 4—Panel B). This was also apparent in alpha diversity comparisons, where shotgun sequencing was associated with significantly lower richness across all sample types (*p* < 0.05), detecting on average 60% fewer AMR gene groups compared to TE sequencing. Shannon’s diversity was also significantly reduced in shotgun samples for all host types except avian samples, likely reflecting the loss of rare taxa below the detection threshold of standard shotgun sequencing (Figure 5). To confirm that these differences were not attributable to the greater sequencing depth of TE libraries, alpha diversity analyses were repeated after rarefying all libraries to a common depth of 6,642 AMR gene alignments, corresponding to the lowest depth among shotgun libraries. TE libraries retained significantly higher richness and Shannon diversity than shotgun libraries in all host groups under this conservative condition (Supplementary Figure S1), indicating that the detection advantage of target enrichment is not an artifact of sequencing depth. These findings underscore the importance of method selection when comprehensive resistome characterization is the goal, as conclusions about resistome diversity drawn from shotgun data alone may underestimate the true complexity of AMR dynamics.

### Resistome diversity differed by library preparation kit but not DNA input

In Part A samples, there were no significant differences in richness or evenness by input DNA amount in any host type. However, samples processed with the Agilent XT kit had higher ARG group-level richness than XT HS in avian (*p* = 0.009), bovine (*p* = 0.024), human (*p* = 0.009), and porcine samples (*p* < 0.001) (Figure 5). Shannon’s diversity was also significantly higher in XT kit samples in avian samples (*p* = 0.03) and in human wastewater (*p* < 0.001). Shannon’s diversity values varied within a narrow range (4.04 to 4.93; mean: 4.54, standard deviation: 0.27). Therefore, significant changes between sample groups should be interpreted with caution and within the context that evenness was largely similar among samples in this study.

### Resistome composition was dominated by tetracycline resistance and clustered by host species

**Figure 6:**
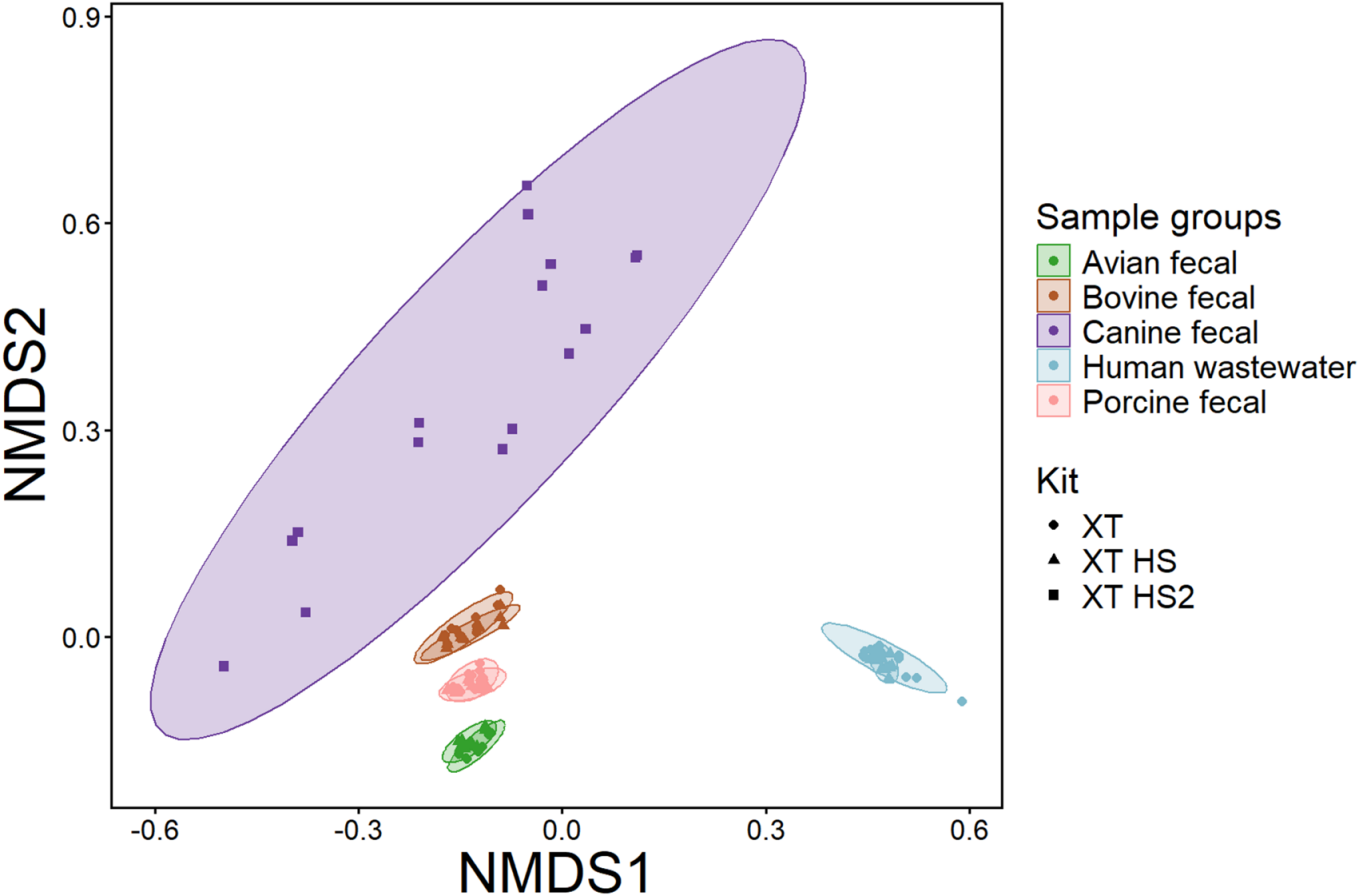
Ordination of resistome composition using non-metric multidimensional scaling (NMDS) of Euclidean distances of Hellinger-transformed, CSS-normalized counts at the AMR group level for all Part A and Part B TE samples. Sample points are colored by host species and shaped by library preparation kit. Samples cluster strongly by host species (PERMANOVA *R*^2^ = 0.88, *p* < 0.001), while kit effects were smaller but significant within individual host groups. Ellipses represent 95% confidence intervals around group centroids. Statistical analysis was performed for Part A and Part B separately for downstream analysis.

**Figure 7:**
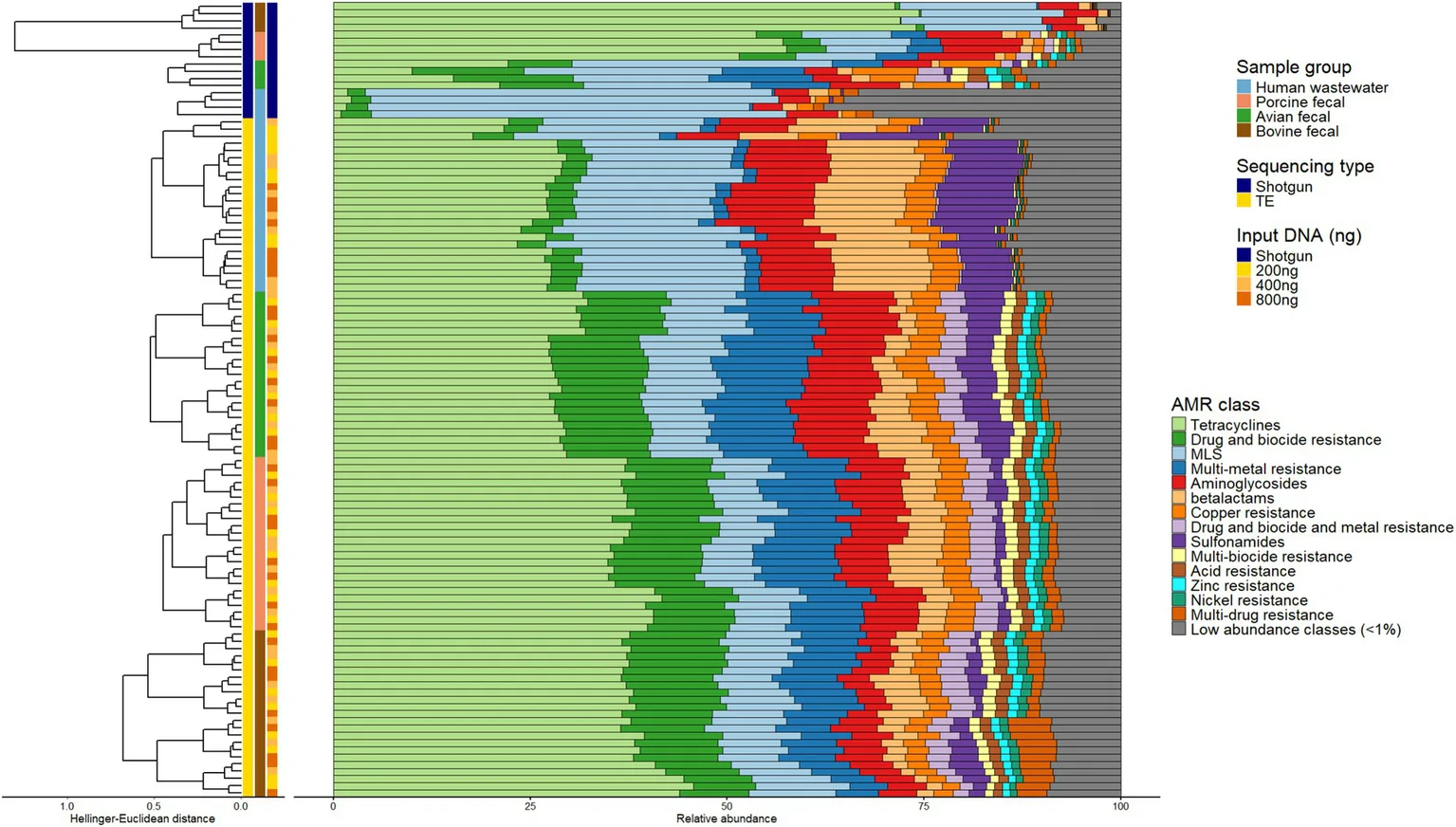
Resistome composition by AMR drug class for Part A samples. Stacked bar plots show the relative abundance of AMR drug classes, with samples ordered by hierarchical clustering (Ward’s linkage on Hellinger-transformed Euclidean distances at the AMR group level). Annotation bars indicate sample group, sequencing type (TE vs. shotgun), and DNA input amount. AMR drug classes with less than 1% median relative abundance across all samples were collapsed into “low-abundance classes (<1%).” Samples cluster primarily by host source regardless of sequencing method, while shotgun samples show reduced compositional diversity than TE libraries despite similar dominant drug class profiles.

**Figure 8:**
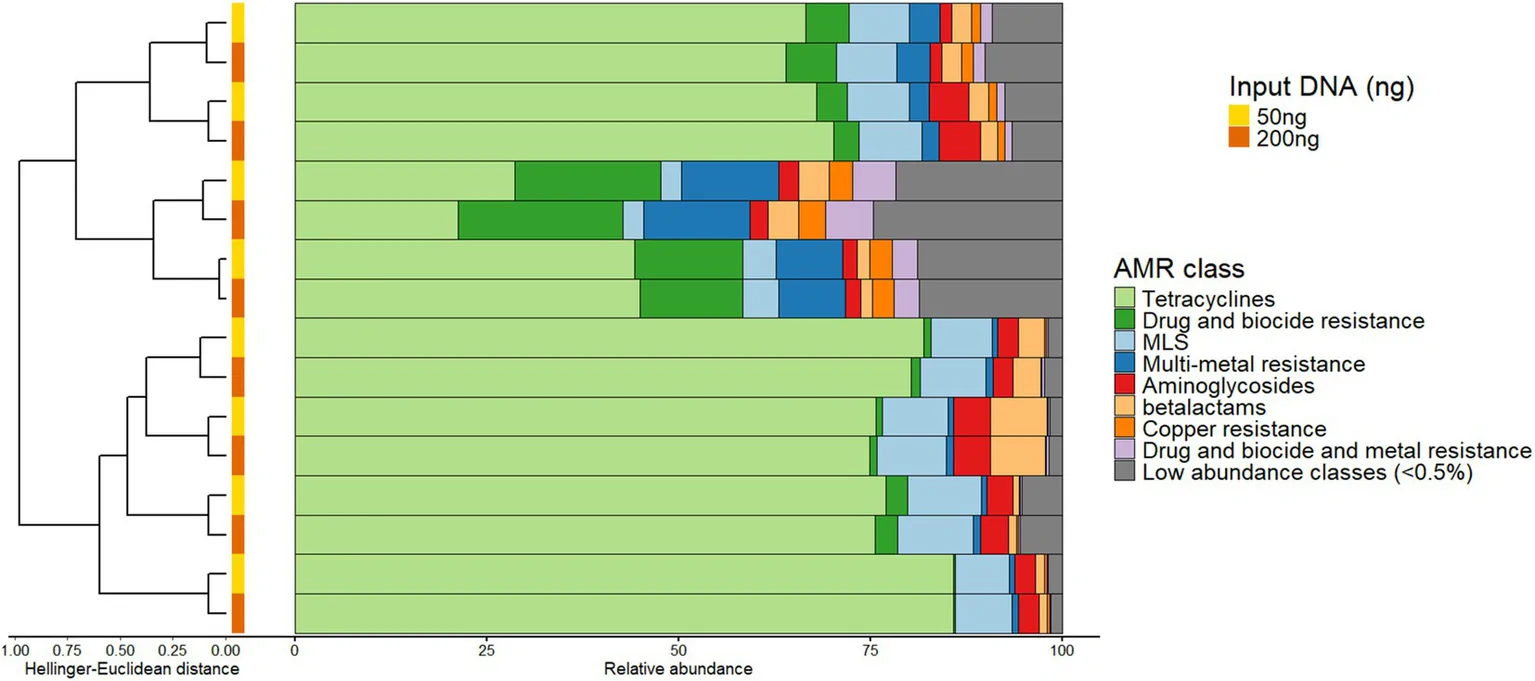
Resistome composition by AMR drug class for Part B canine fecal samples. Stacked bar plots show the relative abundance of AMR drug classes, with samples ordered by hierarchical clustering (Ward’s linkage on Hellinger-transformed Euclidean distances at the AMR group level). The annotation bar indicates DNA input amount (50 ng vs. 200 ng). AMR drug classes with less than 0.5% median relative abundance were collapsed into “low-abundance classes (<0.5%).” No distinct clustering by DNA input amount was observed, consistent with PERMANOVA results (*R*^2^ = 0.002, *p* = 0.493).

Overall, resistome composition in all samples was dominated by resistance to the tetracycline drug class which had a median relative proportion of 35.7% across all samples, compared to drug and biocide resistance (10.6%), macrolide–lincosamide–streptogramin (8.72%), multi-metal resistance (8.47%), and aminoglycosides (7.18%), with all other classes comprising <5% of the relative abundance in samples. The majority of drug classes were found in low abundance, with 38 representing <1% median relative abundance. In Part A, samples were clustered by resistome composition at the AMR group, showing high similarity based on sample host (Figure 6). Shotgun sequencing showed similar patterns in the composition of the dominant taxa, but their lower diversity resulted in significantly different resistome composition (Figure 7). In Part B samples, the resistome composition did not cluster significantly based on input DNA amount (Figure 8).

### Sample source explained most compositional variance; kit and DNA input effects were small and consistent after rarefaction

To evaluate the influence of study variables on the resistome composition of both datasets, excluding shotgun samples, RDA and stepwise model selection were employed, which resulted in the following variables being included in the final model: original sample source, library kit type, and input DNA amount. The RDA model explained 20% of the total variance in the resistome, with the majority constrained by study variables (19.6%) compared to the unconstrained variance (0.4%). The sample source was estimated to have the greatest influence on the resistome, with an estimated 19.3% of the variance explained by this variable compared to library kit type (0.2%) and input DNA amount (0.02%). While major differences were noted in diversity based on sample host species, this variable was dropped from the RDA model during stepwise model selection in favor of the variable representing the original sample ID that was processed in replicate. We tested a custom model including the sample host variable instead of sample source ID and discovered it explained 15.5% of the total variance, emphasizing that host was important in determining the resistome composition, but most of the variance could be explained by the original sample being processed with multiple methods.

An initial marginal PERMANOVA model including both host group and original sample source revealed perfect collinearity between these variables (Df = 0 for the interaction term), indicating that host group membership was entirely determined by original sample identity. Therefore, original sample source was retained as the primary predictor in all subsequent models. Across all Dataset A samples, resistome composition was overwhelmingly driven by original sample source (*R*^2^ = 0.953, *p* < 0.001), while library preparation kit had a small but statistically significant marginal effect (*R*^2^ = 0.015, *p* < 0.001). DNA input amount did not significantly affect resistome composition at the full dataset level (*R*^2^ = 0.001, *p* = 0.060). Within-species analyses revealed consistent patterns across host types (Table 1). Library preparation kit significantly affected resistome composition in bovine (*R*^2^ = 0.134, *p* < 0.001), avian (*R*^2^ = 0.171, *p* < 0.001), and porcine samples (*R*^2^ = 0.212, *p* < 0.001), although in all cases the effect size was small relative to original sample source, which explained 66%–79% of within-species variance. DNA input amount was not a significant predictor of resistome composition in any host species except porcine samples under CSS normalization (*R*^2^ = 0.025, *p* = 0.048), an effect that did not reach significance after rarefaction (*p* = 0.078), suggesting it may be driven by sequencing depth variation rather than true compositional differences. Human wastewater samples were the exception to the overall pattern: Library kit was significantly associated with resistome composition (*R*^2^ = 0.259, *p* < 0.001); however, the dispersion test was also significant (*p* = 0.01), indicating that differences in within-group variance contributed to this result and that the PERMANOVA finding should be interpreted with caution. Original sample source did not significantly explain resistome composition in human wastewater samples (*R*^2^ = 0.141, *p* = 0.089), which likely reflects the composite nature of wastewater samples and greater inherent within-group variability compared to animal fecal samples.

**Table 1:** PERMANOVA results comparing resistome composition (Hellinger-transformed Euclidean distances at AMR group level) across study variables for CSS-normalized and rarefied data.

Sample set	Library kit type^1^		Input DNA^2^		Sample replicate	
	CSS	Rarefied	CSS	Rarefied	CSS	Rarefied
Dataset A	*R*^2^ = 0.015, *p* < 0.001	*R*^2^ = 0.015, *p* < 0.001	*R*^2^ = 0.001, *p* = 0.06	*R*^2^ = 0.002, *p* = 0.072	*R*^2^ = 0.953, *p* < 0.001*	*R*^2^ = 0.95, *p* < 0.001*
Bovine fecal	*R*^2^ = 0.134, *p* < 0.001	*R*^2^ = 0.135, *p* = 0.009	*R*^2^ = 0.022, *p* = 0.002	*R*^2^ = 0.024, *p* < 0.001	*R*^2^ = 0.791, *p* < 0.001	*R*^2^ = 0.775, *p* < 0.001
Avian fecal	*R*^2^ = 0.171, *p* < 0.001	*R*^2^ = 0.168, *p* < 0.001	*R*^2^ = 0.02, *p* = 0.995	*R*^2^ = 0.016, *p* = 0.195	*R*^2^ = 0.66, *p* < 0.001	*R*^2^ = 0.704, *p* < 0.001
Porcine fecal	*R*^2^ = 0.212, *p* < 0.001	*R*^2^ = 0.206, *p* < 0.001	*R*^2^ = 0.025, *p* = 0.048	*R*^2^ = 0.027, *p* = 0.078	*R*^2^ = 0.66, *p* < 0.001	*R*^2^ = 0.639, *p* < 0.001
Human wastewater	*R*^2^ = 0.259, *p* < 0.001*	*R*^2^ = 0.249, *p* < 0.001*	*R*^2^ = 0.11, *p* = 0.058	*R*^2^ = 0.11, *p* = 0.211	*R*^2^ = 0.141, *p* = 0.089	*R*^2^ = 0.141, *p* = 0.086
Dataset B (canine fecal)	—	—	*R*^2^ = 0.002, *p* = 0.493	*R*^2^ = 0.002, *p* = 0.511	*R*^2^ = 0.98, *p* < 0.001	*R*^2^ = 0.98, *p* < 0.001

^1^SureSelect XT vs. SureSelect XTHS. ^2^Dataset A: 800 ng, 400 ng or 200 ng; Dataset B: 200 ng or 50 ng. *R*^2^ values represent the proportion of variance explained by each factor under a marginal model (adonis2, by = “margin,” 9,999 permutations). Tests were performed on all Dataset A target-enriched samples combined and within each host species separately, with per-species distances extracted from the full Dataset A distance matrix. Dataset B (canine) was analyzed independently. Significant *p*-values (<0.05) are shown in bold. *Dispersion test (PERMDISP) was also significant for this comparison (*p* < 0.05); the PERMANOVA result should be interpreted with caution as it may partially reflect differences in within-group variance rather than centroid differences.

In Dataset B canine fecal samples, resistome composition was strongly driven by original sample source (*R*^2^ = 0.980, *p* < 0.001), while DNA input amount had no significant effect (*R*^2^ = 0.002, *p* = 0.493), confirming that TE library preparation produces consistent resistome profiles even with substantially reduced DNA input (50 ng vs. 200 ng). The results were highly consistent between CSS-normalized and rarefied analyses across all comparisons (Table 1), indicating that observed patterns were not substantially driven by differences in sequencing depth.

### Kit-associated differential abundance was largely restricted to a few low-abundance drug classes

**Figure 9:**
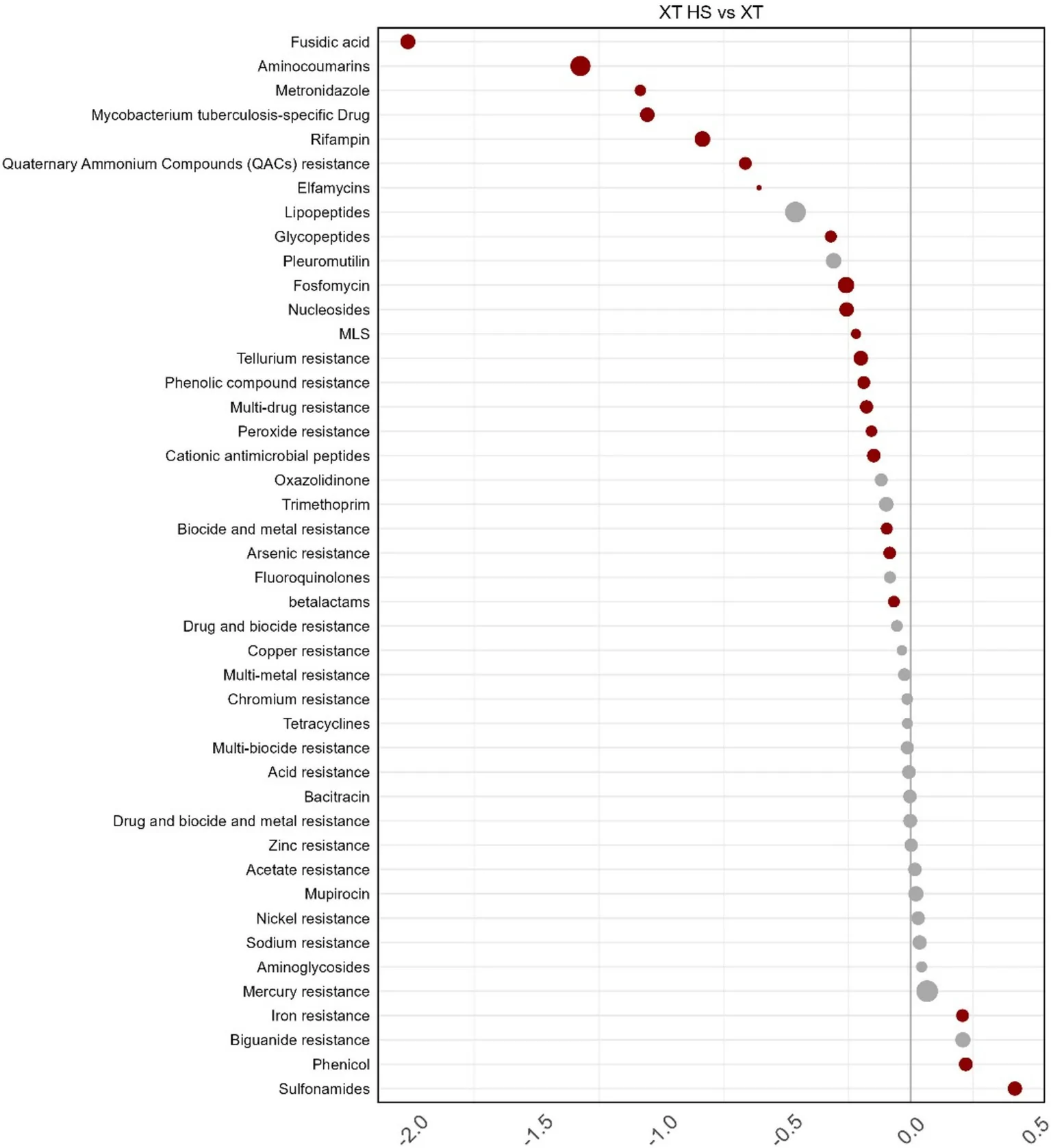
Differential abundance of AMR drug classes between library preparation kits. ANCOMBC2 results comparing AMR class abundance between SureSelect XT-HS and XT kit samples, controlling for sample host, DNA input amount, and original sample source. Log fold change (logFC) is shown on the *x*-axis; positive values indicate enrichment in XT-HS samples. Significant differences (adjusted *p* < 0.05) are shown in red. Circle size corresponds to the bias-adjusted mean abundance of each AMR class in XT-HS samples. The majority of differentially abundant classes (19/22) were depleted in XT-HS samples, although most changes were of low magnitude and in low-abundance classes.

An ANCOMBC model was trained on Part A samples to assess for differentially abundant AMR classes based on library kit type, while controlling for sample host, input DNA amount, and original sample source. In all, the ANCOMBC model identified 22/44 differentially abundant classes between samples processed with the XT and XT HS kit (Figure 9). Of the differentially abundant classes, the majority (19/22) were depleted in samples with the XT HS kit, although only four classes had a log fold change greater than −1: fusidic acid (logFC = −2.016, *p* < 0.001), aminocoumarins (logFC = −1.324, *p* < 0.001), metronidazole (logFC = −1.084, *p* < 0.001), and *Mycobacterium tuberculosis*-specific drug resistance (logFC = −1.056, *p* < 0.001). Alternatively, only three AMR classes were significantly enriched in XT HS samples: iron resistance (logFC = 0.028, *p* < 0.001), phenicol resistance (logFC = 0.221, *p* < 0.001), and sulfonamides (logFC = 0.418, *p* < 0.001). Of the AMR classes listed above, it is important to note that sulfonamides were present at a median relative abundance of 2.52% across all samples, while the remaining classes were present at less than 1% median relative abundance.

## Discussion

### Target-enriched library preparation kits yielded comparable resistome profiles

Although library preparation protocols differ, TE sequencing produced highly comparable results for resistome profiles across kits and DNA input amounts. Variation in major features for the resistome composition was driven primarily by sample source, with only modest effects attributable to library kit. Samples clustered most strongly by host origin and original sample identity, even when processed in replicate using different kits and DNA input amounts. While the SureSelect XT kit identified a greater number of AMR gene groups, differences between kits were small relative to host-associated variation and were largely confined to low-abundance features. Importantly, the XT-HS kit enabled successful analysis of samples with limited DNA input while maintaining comparable resistome structure. Together, these findings indicate that either library preparation approach can be used effectively for target-enriched resistome sequencing, provided that a single kit is applied consistently across all samples within a study. However, comparisons across studies using different kits should consider the potential influence of low-abundance and sparse features.

### Target enrichment substantially improves detection of low-abundance ARGs relative to shotgun sequencing

Consistent with previous reports (Noyes et al., 2017; Guitor et al., 2019), there was a substantial improvement in resistome detection achieved by TE sequencing relative to standard shotgun metagenomics. TE sequencing detected 12-fold more unique AMR gene groups and achieved a 7.9-fold increase in on-target sequencing proportions when compared using the same samples. This improvement was observed across all host species, although the magnitude varied; for example, in human wastewater samples, on-target proportions increased from 0.04% with shotgun sequencing to 1.0% when using TE. These results reinforce findings that shotgun metagenomics may underrepresent rare or low-abundance resistance determinants (Noyes et al., 2017; Guitor et al., 2019). Additionally, TE sequencing provides a more sensitive and comprehensive assessment, particularly in complex microbial communities or samples with high amounts of off-target DNA, such as host. The lack of a significant original sample source effect in human wastewater (*R*^2^ = 0.141, *p* = 0.089) contrasts sharply with the strong sample-level clustering observed in all animal fecal groups (*R*^2^ = 0.66–0.98) and likely reflects the composite and highly variable nature of wastewater rather than a methodological limitation. This distinction underscores the importance of considering sample matrix characteristics when interpreting resistome comparisons.

### Biological source, not laboratory protocol, is the dominant driver of resistome composition

The primary determinant of resistome composition in this study was the original sample source, which explained 19.3% of total variance, with host-associated differences accounting for substantially more variation than library preparation kit (0.2%) or DNA input amount (0.02%). Although statistically detectable differences between kits were observed within some host groups, these effects were modest in magnitude and largely restricted to low-abundance resistance classes, as highlighted by the ANCOM-BC analysis results. Relative to the differences among host species and among original samples, which explained 66%–79% of within-species variance, kit-associated differences were small, and samples processed with different kits still clustered by biological origin rather than by kit. We, therefore, interpret these results as indicating that either kit can support valid within-study comparisons of resistome composition, provided the kit is applied uniformly across samples. We do not interpret them as evidence that kit choice is biologically unimportant, and we note specifically that comparisons of absolute richness, of low-abundance drug classes, or of data pooled across studies using different kits are likely to be affected. The consistency of kit-associated effect sizes across CSS-normalized and rarefied analyses indicates these differences are reproducible rather than artifacts of normalization or sequencing depth.

RDA and PERMANOVA were used together because they offer complementary strengths. PERMANOVA is a non-parametric, distance-based test that makes no assumptions about the shape of relationships between variables and community composition, making it well-suited for detecting and quantifying group differences in complex datasets. RDA, in contrast, is a constrained ordination that assumes linear relationships but provides a visual representation of how study variables relate to overall compositional patterns and allows stepwise variable selection to identify which predictors contribute independently. The quantitative R^2^ values from the two methods are not directly comparable because they partition variance differently; PERMANOVA operates on pairwise distances while RDA decomposes multivariate inertia, but both approaches consistently identified original sample source as the dominant driver of resistome composition, with library kit and DNA input contributing minimally.

Detailed biological comparison of these sample types was not an aim of this study and has been reported previously (Noyes et al., 2017), but the pattern observed here is notable: Human wastewater had the highest on-target proportions among TE libraries and the lowest among shotgun libraries generated from the same sample material. As a composite matrix aggregating waste from many individuals in a wider region, wastewater may harbor a greater diversity of ARGs available for capture, each at low relative abundance which is precisely the condition under which enrichment offers the greatest gain over shotgun sequencing. Additionally, the composition of the reference database may also contribute, as the MEGARich probe set was designed from MEGARes, a compilation of published ARG sequences weighted toward determinants described in clinical and human-associated isolates. Theoretically, this could improve both probe hybridization and subsequent alignment for human-associated communities. Apparent differences in enrichment efficiency between sample types should therefore be interpreted as a joint property of the sample matrix and the database from which the probes were derived, rather than as a direct measure of resistome abundance.

### DNA input can be reduced without compromising resistome characterization

From a practical perspective, these findings provide guidance for the design of resistome studies using target-enriched sequencing. The consistency of the results across DNA input amounts suggests that reliable resistome characterization can be achieved even when DNA availability is limited, expanding the applicability of this approach to low-biomass or archival samples. Similarly, the comparable performance of library preparation kits indicates that researchers can select workflows based on logistical considerations such as cost, throughput, and available DNA without substantially affecting biological interpretation. However, caution is warranted when directly comparing low-abundance or sparsely distributed resistance features when data were generated using different enrichment kits or library preparation protocols.

This study has limitations that should be considered when interpreting the findings. The number of samples within each host group was modest, and the design was not fully balanced across all combinations of host species, library preparation kits, and DNA input amounts (Figure 1). As a result, subtle interactions between sample type and laboratory workflow may not have been fully captured. The use of replicate processing of identical source samples allowed direct evaluation of methodological effects independent of biological variation. Although probe-based enrichment tolerates some sequence mismatch, target-enriched sequencing is inherently limited to genes represented in the probe set design and may not detect highly divergent or novel resistance determinants. Performance was also not evaluated across a wide range of sample matrices that may have influenced enrichment efficiency, particularly in samples with high proportions of off-target DNA. TE and shotgun libraries were not sequenced to equal depth (mean 30.5 vs. 18.0 million paired reads). Because on-target proportion is a ratio, the enrichment estimate is unaffected by this difference, and richness comparisons were additionally confirmed after rarefying to equal alignment depth (Supplementary Figure S1). We note that this rarefaction was applied to on-target alignment counts rather than by subsampling raw sequencing reads; therefore, it removes the enrichment advantage of TE libraries and constitutes a conservative test of whether target enrichment recovers greater resistome diversity per on-target read. Additionally, while rarefaction to the minimum sequencing depth (127,467 reads) was used in TE comparisons as a sensitivity analysis and produced highly concordant results, this depth represents a relatively conservative threshold, and the consistency observed here may not extend to studies with more extreme variation in sequencing depth or substantially lower minimum depths. Despite these limitations, the consistent patterns observed across diverse sample types support the generalizability of the findings. Our comparison was also restricted to a single target-enrichment chemistry (Agilent SureSelect) and to library preparation kits within that system (XT, XT-HS, and XT-HS2). Other enrichment platforms and library construction chemistries are in common use, and we did not evaluate whether concordance extends across vendors or capture systems. Cross-vendor differences would plausibly be larger than the within-system differences observed here because probe design, hybridization chemistry, and adapter and library construction all differ between platforms. The results should therefore be read as evidence that resistome profiles are robust to the variables we examined, DNA input amount and library preparation kit within the SureSelect system, rather than as a general claim of equivalence across all enrichment technologies. Direct comparison across enrichment platforms remains an important direction for future work.

In summary, TE sequencing substantially improves detection of ARGs compared to standard shotgun sequencing while producing consistent characterization of resistome features across library preparation protocols and DNA input amounts. This is particularly important for the detection and representation of rare or low-abundance AMR determinants. Variation in resistome composition was driven primarily by biological differences among samples rather than methodological factors, supporting the robustness of this approach for comparative studies. Together, these findings demonstrate that TE sequencing provides a flexible, reliable, and cost-effective approach for comprehensive resistome characterization that dramatically improves detection sensitivity and decreases the need for excessive sequencing depth.

## Data Availability

The datasets (count matrices) and metadata analyzed in this study can be found in the article/Supplementary material. R scripts used in these analyses are publicly available at DOI: 10.5281/zenodo.22087968. Raw sequencing data generated in this study have been deposited in NCBI under BioProject PRJNA1441486. Shotgun metagenomic sequences were obtained from a previously published dataset (BioProject PRJNA339554; accessions SRR4425656 and SRR4425681–SRR4425696).

## Footnotes

1
https://www.meglab.org/megares

2
https://github.com/Isabella136/AmrPlusPlus_SNP
