# Supplementary material for: Different DNA input amounts and library preparation kits yield comparable resistome characterization using target-enriched metagenomic sequencing
Source: Front Microbiol. 2026 Sep 10;17:1907850. doi: 10.3389/fmicb.2026.1907850 (PMC13601221; doi:10.3389/fmicb.2026.1907850)

SUPPLEMENTAL FIGURE

Figure S1: Alpha diversity of the resistome after rarefaction to equal sequencing depth. To evaluate whether differences in detection between sequencing methods were attributable to differences in sequencing depth, resistome count matrices from target-enriched and shotgun libraries were rarefied to a common depth of 6,642 AMR gene alignments, corresponding to the lowest depth observed among shotgun libraries, using rarefy_even_depth() (phyloseq; sampling without replacement, seed = 42). All samples were retained at this depth. A) Boxplot of unique observed ARG groups (richness) on the y-axis, grouped by sample host source and library type. B) Boxplot of Shannon's diversity on the y-axis, grouped by sample host source and library kit type. Letters above the boxplots indicate the results of pairwise Wilcoxon rank-sum tests with Benjamini-Hochberg correction for multiple comparisons, performed within each sample group. Groups that share a letter are not significantly different (adjusted $P \geq 0.05$); groups that share no letter differ significantly (adjusted $P < 0.05$). Note that rarefaction was applied to on-target alignment counts rather than to raw sequencing reads, and therefore removes the enrichment advantage of target-enriched libraries; this represents a conservative test of whether target enrichment recovers greater resistome diversity per on-target read. Patterns were consistent with those observed in the unrarefied data (Figure 5).

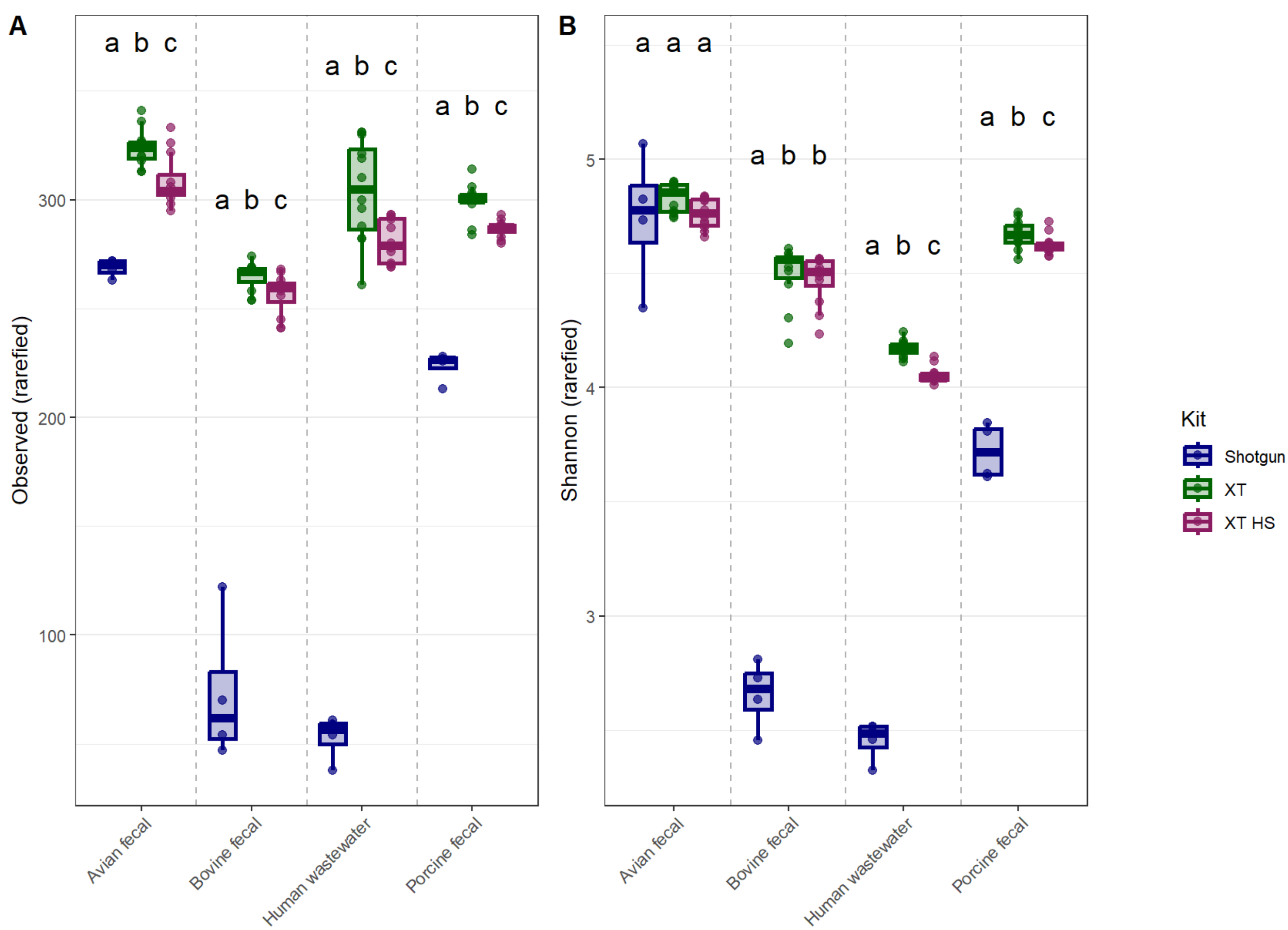

Supplement: Supplementary file 1 [file Image_1.pdf]
